# Supplementary material for: Lantibiotics Produced by Oral Inhabitants as a Trigger for Dysbiosis of Human Intestinal Microbiota
Source: Int J Mol Sci. 2021 Mar 25;22(7):3343. doi: 10.3390/ijms22073343 (PMC8037337; doi:10.3390/ijms22073343)
Supplement: Supplementary file 1 [file ijms-22-03343-s001.pdf]

Figure S1. Principal-coordinate analysis (PCoA) of the fecal microbiota. Plots of PCoA between the lantibiotic positive (red square, group 1), or negative subjects (blue circle, group 2) were performed based on weighted UniFrac (a), unweighted UniFrac (b), or Bray-Curtis (c) distances of the mouse fecal bacterial communities.

Figure S2. Microbial taxonomic change of the fecal microbiota between lantibiotic positive and negative subjects at phylum level. Relative abundance based on OTUs in Lentisphaerae (a), Actinobacteria (b), Bacteroidetes (c), or Proteobacteria (d) of the fecal samples between groups.

Figure S3. Comparison of the fecal microbial diversity and microbial taxonomic change between Mutacin I/II positive (group 1a), Smb positive (group 1b) and both negative (group 2) groups. Chao 1 diversity (a) and Shannon diversity (b) based on operational taxonomic units (OTUs). (c) Comparison of relative abundance of OTUs in bacterial composition of the fecal samples at phylum level between groups. Relative abundance based on OTUs in Actinobacteria (d), Firmicutes (e), Bacteroidetes (f), or Proteobacteria (g) of the fecal samples between groups. Relative abundance based on OTUs in genus *Anaerostipes* (h), *Ruminococcaceae* (i), *Selenomonas* (j) or *Bulleidia* (k) of the fecal samples between groups. All of the data represent measures for 5 subjects (Mutacin I/III positive group: Group 1a), 8 subjects (Smb positive group: Group 2b) and 56 subjects (the lantibiotic negative group: Group 2). All of the boxplots for each group represent the interquartile range (25% - 75%) and the line within the box represents the median value. Kruskal-Wallis H-test was used to test for significant differences among sample distances and Mann-Whitney U-test was then used for significant differences between groups. Asterisks show significant differences ($p<0.05$).

Figure S4. Comparison of saliva microbial taxonomic change between lantibiotic positive and negative groups. Chao 1(a) and Shannon diversity (b) based on operational taxonomic units (OTUs). Principal-coordinate analysis (PCoA) of the saliva microbiota between the lantibiotic positive (red square), and negative groups (blue circle) was performed based on weighted UniFrac (c), unweighted UniFrac (d), or Bray-Curtis (e) distances of the mouse fecal bacterial communities. (f) Comparison of relative abundance of OTUs in bacterial composition of the saliva samples at phylum level between groups. Relative abundance based on OTUs (g) or quantitative Real-time PCR (h) in Firmicutes of the saliva samples between groups. Relative abundance based on OTUs in Proteobacteria (i), Actinobacteria (j), or Bacteroidetes (k). Relative abundance based on

OTUs in genus *Porphylomonas* (l), [*Prevotella*] (m), or *Dorea* (n) of the saliva samples between groups. All of the boxplots for each group represent the interquartile range (25% - 75%) and the line within the box represents the median value. Mann-Whitney U-test was used to test for significant differences between sample distances and asterisks show significant differences ($p<0.05$).

Figure S5. Comparison of the fecal microbial diversity and composition change in mice fed with lantibiotics. Chao 1(a) and Shannon diversity (b) in the mice fed with *S. mutans* culture supernatants containing Mutacin I (group 3; n=10), supernatants of Mutacin I mutant S. mutans (group 4; n = 10) and medium only group (group 5; n = 10) based on operational taxonomic units (OTUs). Relative abundance based on OTUs in family *Lachnospiraceae* (c) or *Prevotella* (d) of the mouse fecal samples between groups. All of the boxplots for each group represent the interquartile range (25% - 75%) and the line within the box represents the median value. Kruskal-Wallis H-test was used to test for significant differences among sample distances and Mann-Whitney U-test was then used for significant differences between groups. Asterisks show significant differences ($p<0.05$).

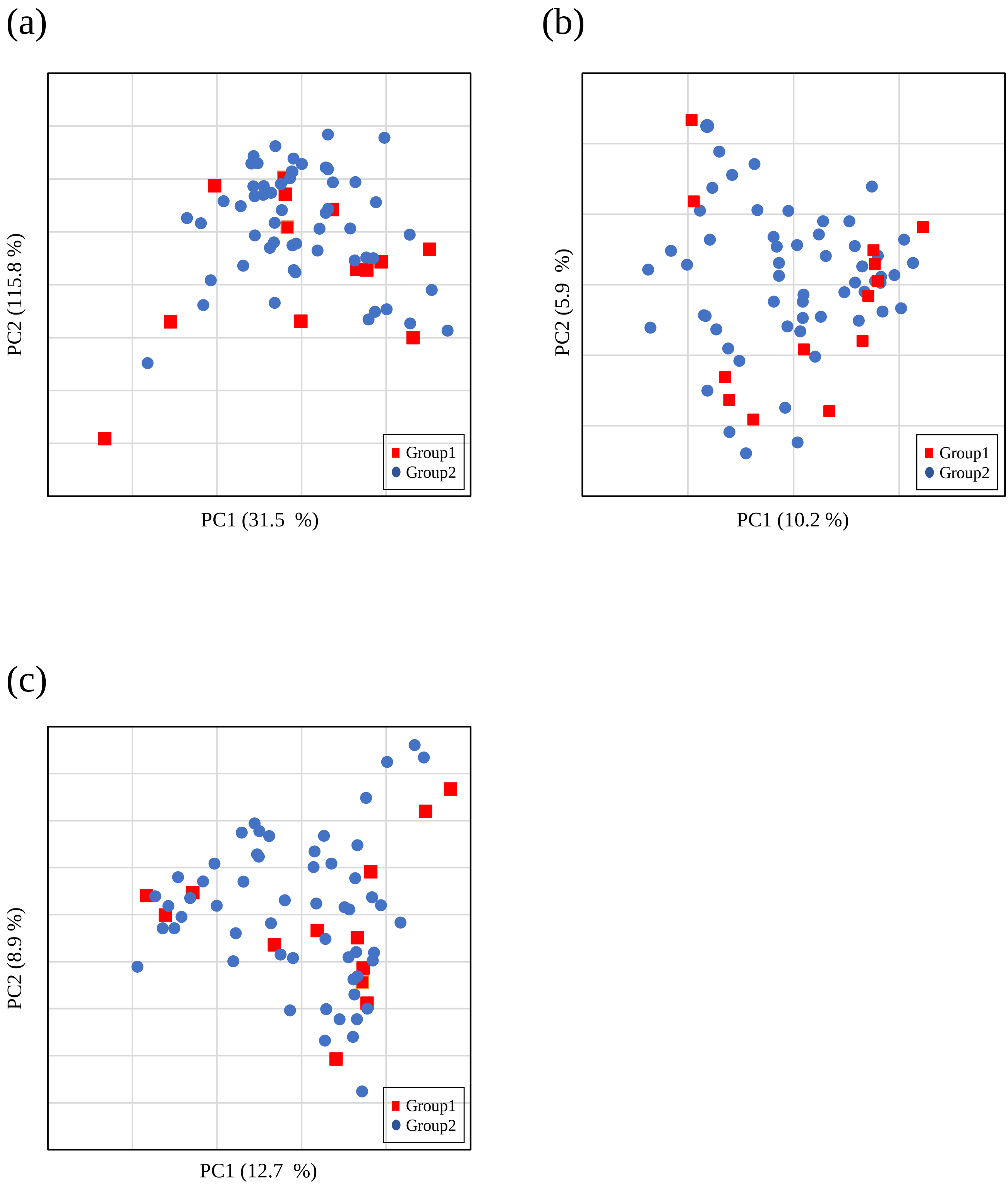
(a)
PC2 (115.8 %)
PC1 (31.5 %)
Group1
Group2
(b)
PC2 (5.9 %)
PC1 (10.2 %)
Group1
Group2
(c)
PC2 (8.9 %)
PC1 (12.7 %)
Group1
Group2

Figure S1

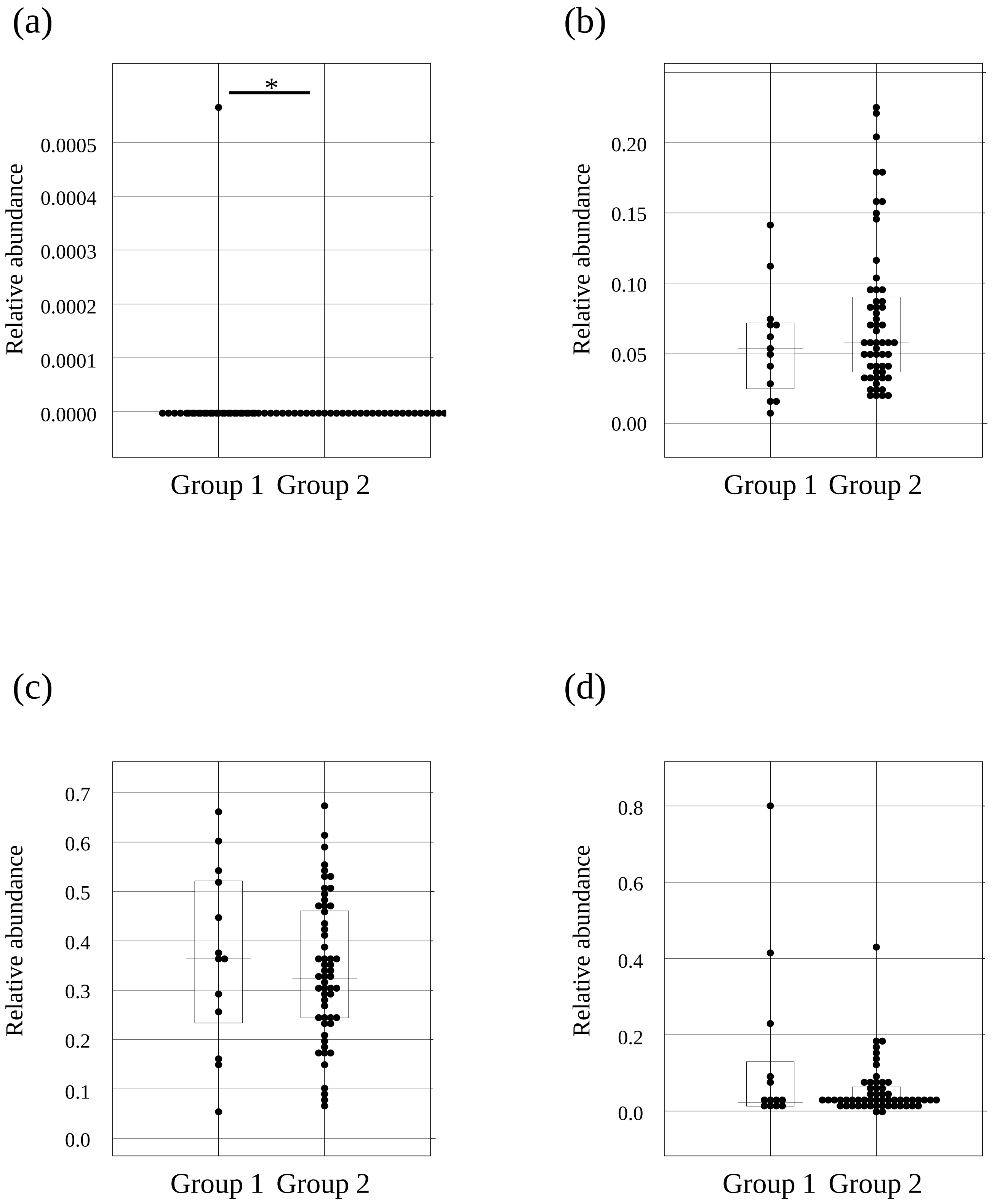
(a)
*
0.0005
0.0004
0.0003
0.0002
0.0001
0.0000
Relative abundance
Group 1
Group 2
(b)
0.20
0.15
0.10
0.05
0.00
Relative abundance
Group 1
Group 2
(c)
0.7
0.6
0.5
0.4
0.3
0.2
0.1
0.0
Relative abundance
Group 1
Group 2
(d)
0.8
0.6
0.4
0.2
0.0
Relative abundance
Group 1
Group 2

Figure S2

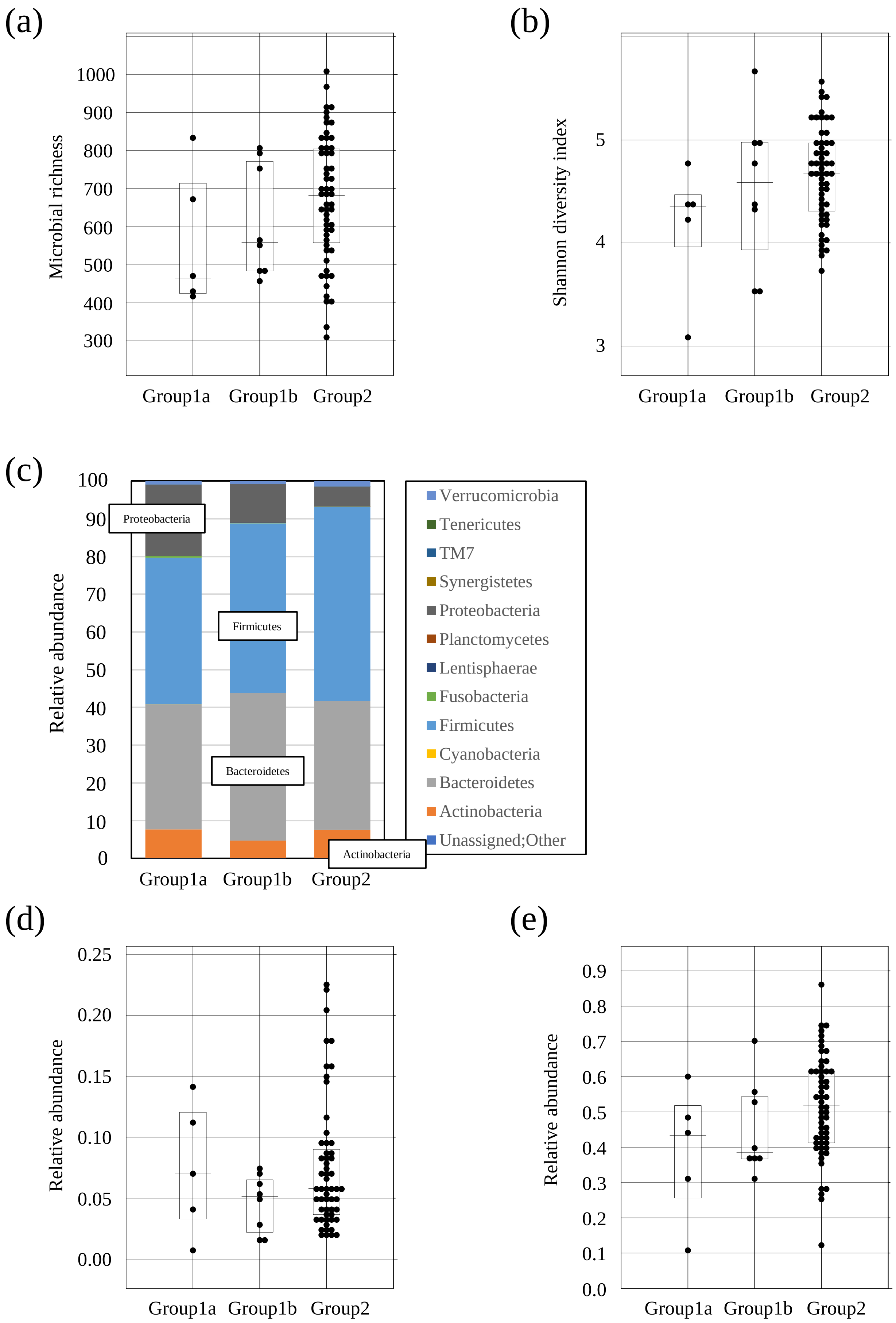
(a)
Microbial richness
1000
900
800
700
600
500
400
300
Group1a
Group1b
Group2
(b)
Shannon diversity index
5
4
3
Group1a
Group1b
Group2
(c)
Relative abundance
100
90
80
70
60
50
40
30
20
10
0
Proteobacteria
Firmicutes
Bacteroidetes
Actinobacteria
Group1a
Group1b
Group2
Verrucomicrobia
Tenericutes
TM7
Synergistetes
Proteobacteria
Planctomycetes
Lentisphaerae
Fusobacteria
Firmicutes
Cyanobacteria
Bacteroidetes
Actinobacteria
Unassigned;Other
(d)
Relative abundance
0.25
0.20
0.15
0.10
0.05
0.00
Group1a
Group1b
Group2
(e)
Relative abundance
0.9
0.8
0.7
0.6
0.5
0.4
0.3
0.2
0.1
0.0
Group1a
Group1b
Group2

Figure S3-1

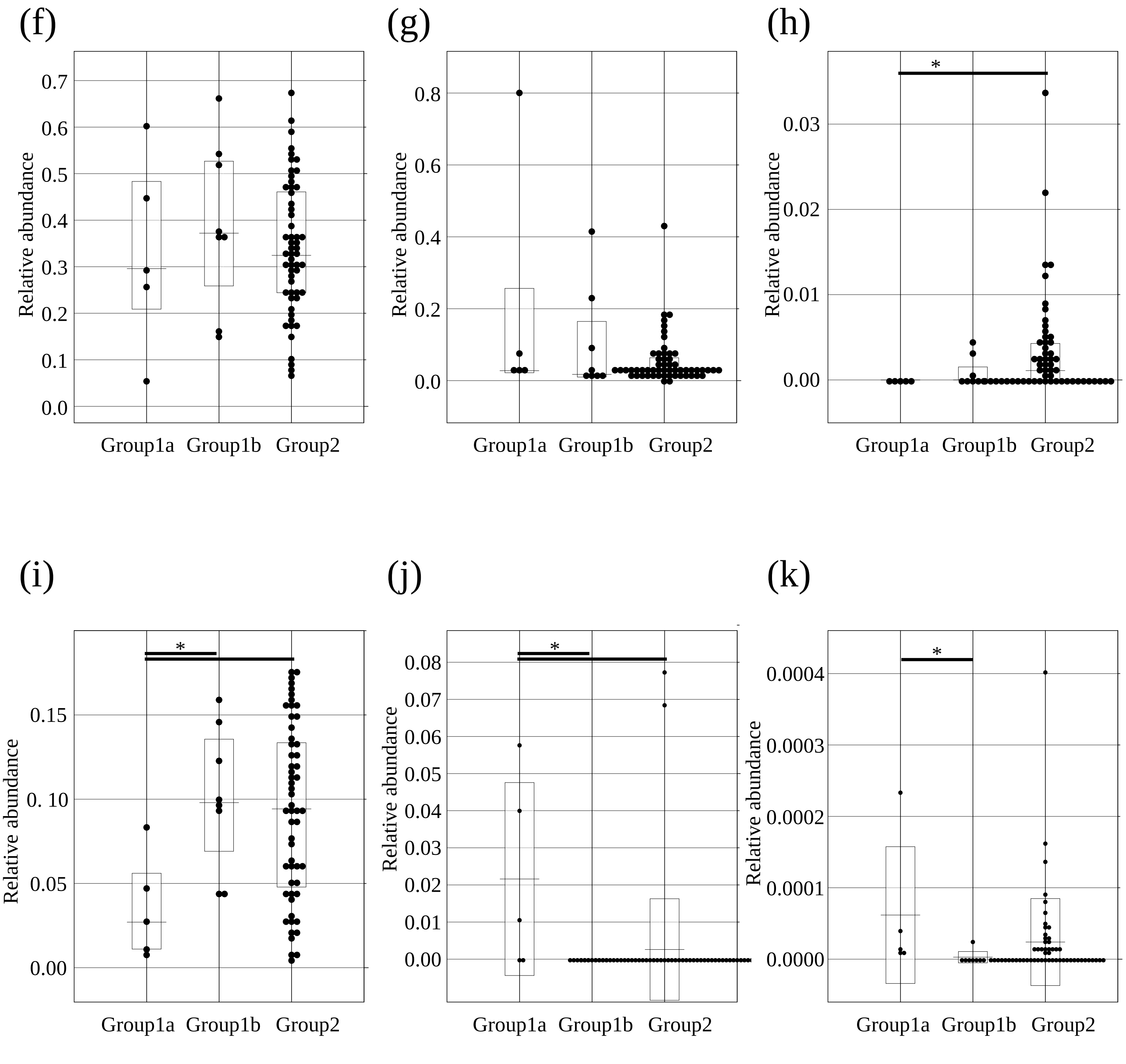

(f)
Relative abundance
0.7
0.6
0.5
0.4
0.3
0.2
0.1
0.0
Group1a Group1b Group2
(g)
Relative abundance
0.8
0.6
0.4
0.2
0.0
Group1a Group1b Group2
(h)
*
Relative abundance
0.03
0.02
0.01
0.00
Group1a Group1b Group2
(i)
*
Relative abundance
0.15
0. 10
0.05
0.00
Group1a Group1b Group2
(j)
*
Relative abundance
0.08
0.07
0.06
0.05
0.04
0.03
0.02
0.01
0.00
Group1a Group1b Group2
(k)
*
Relative abundance
0.0004
0.0003
0.0002
0.0001
0.0000
Group1a Group1b Group2

Figure S3-2

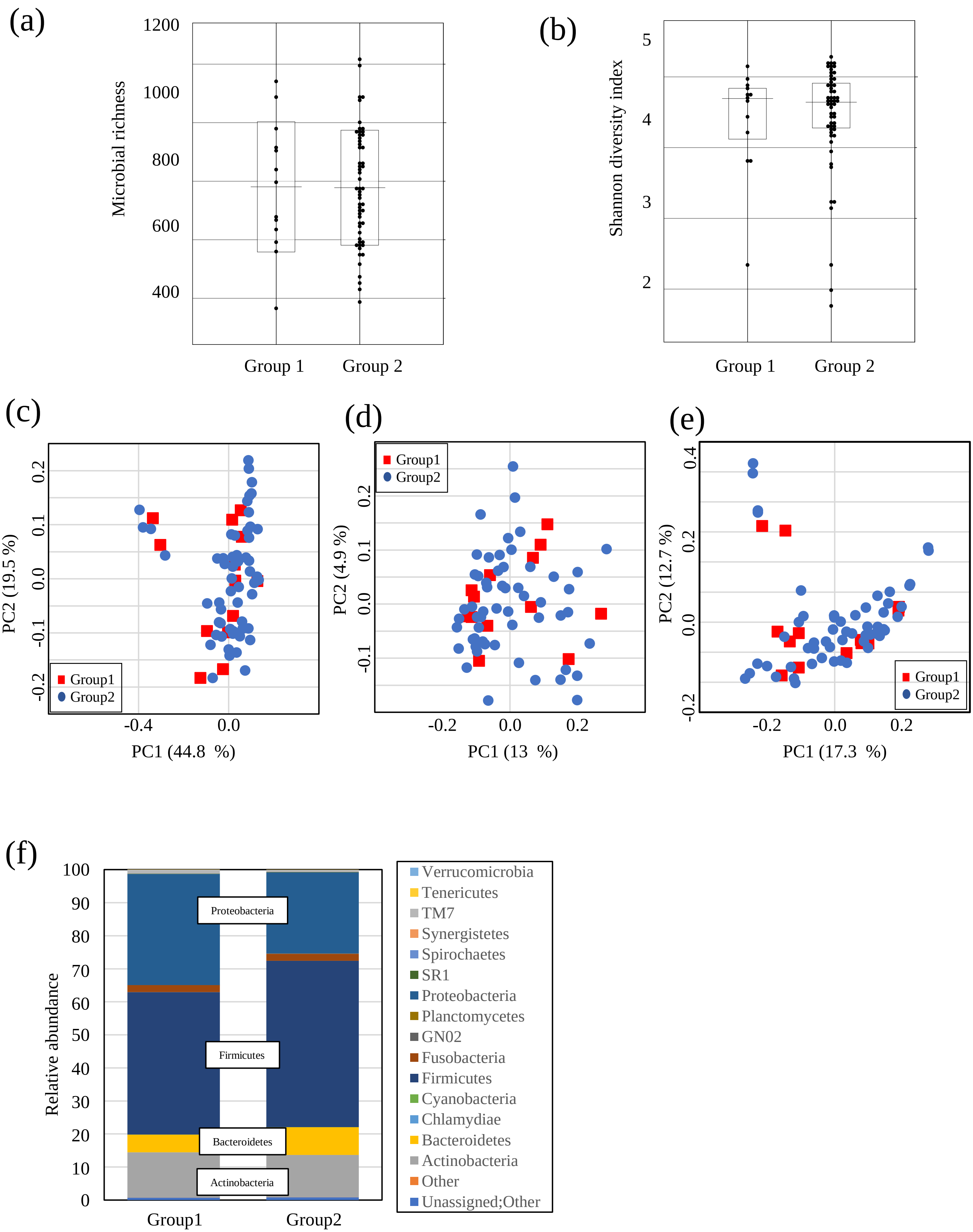
(a)
Microbial richness
1200
1000
800
600
400
Group 1
Group 2
(b)
Shannon diversity index
5
4
3
2
Group 1
Group 2
(c)
PC2 (19.5 %)
PC1 (44.8 %)
Group1
Group2
(d)
PC2 (4.9 %)
PC1 (13 %)
Group1
Group2
(e)
PC2 (12.7 %)
PC1 (17.3 %)
Group1
Group2
(f)
Relative abundance
Proteobacteria
Firmicutes
Bacteroidetes
Actinobacteria
Group1
Group2
Verrucomicrobia
Tenericutes
TM7
Synergistetes
Spirochaetes
SR1
Proteobacteria
Planctomycetes
GN02
Fusobacteria
Firmicutes
Cyanobacteria
Chlamydiae
Bacteroidetes
Actinobacteria
Other
Unassigned;Other

Figure S4-1

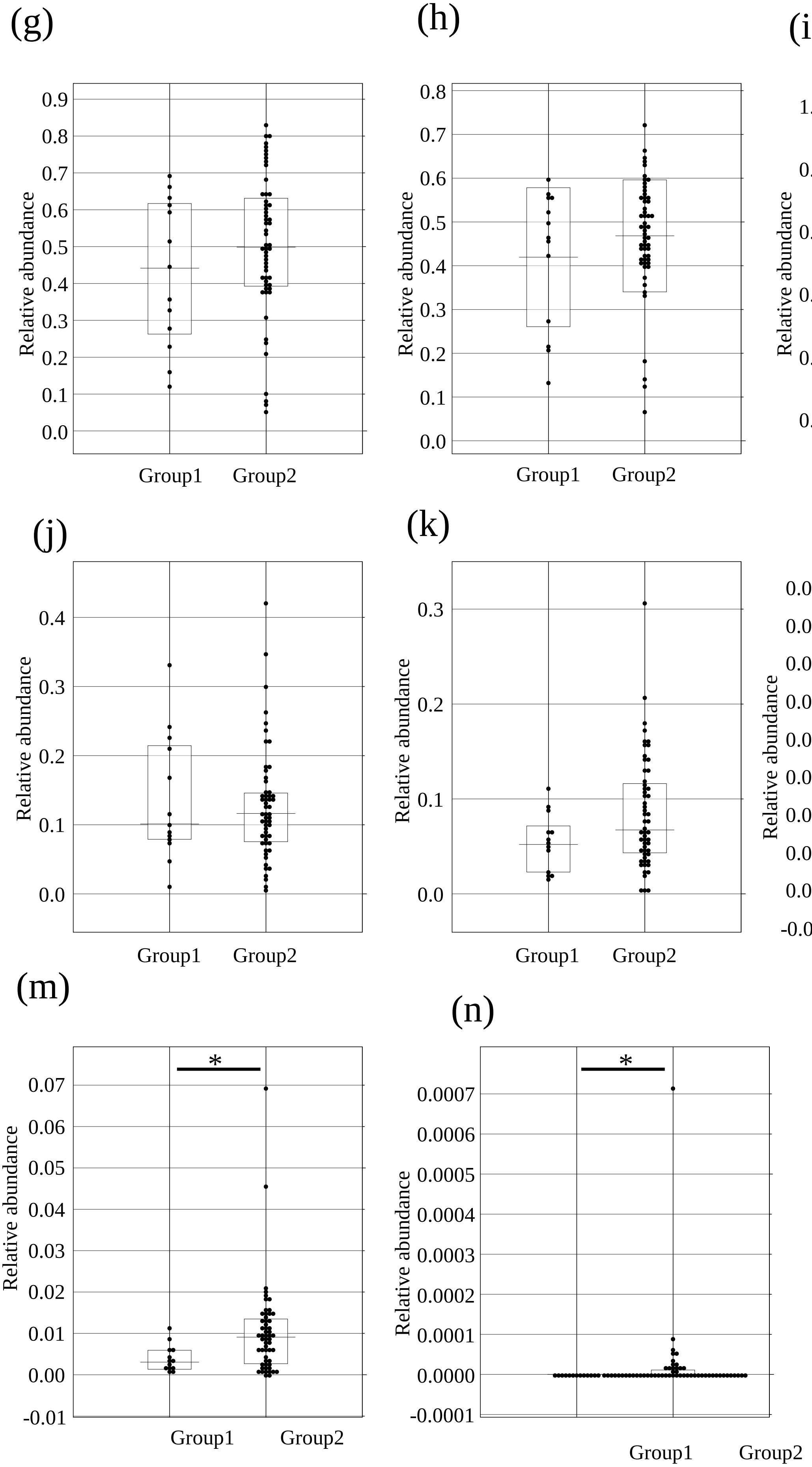
(g)
(h)
(i)
(j)
(k)
(l)
(m)
(n)
Relative abundance
Group1
Group2
0.9
0.8
0.7
0.6
0.5
0.4
0.3
0.2
0.1
0.0
1.0
0.08
0.07
0.06
0.05
0.04
0.03
0.02
0.01
0.00
-0.01
0.0007
0.0006
0.0005
0.0004
0.0003
0.0002
0.0001
0.0000
-0.0001
*

Figure S4-2

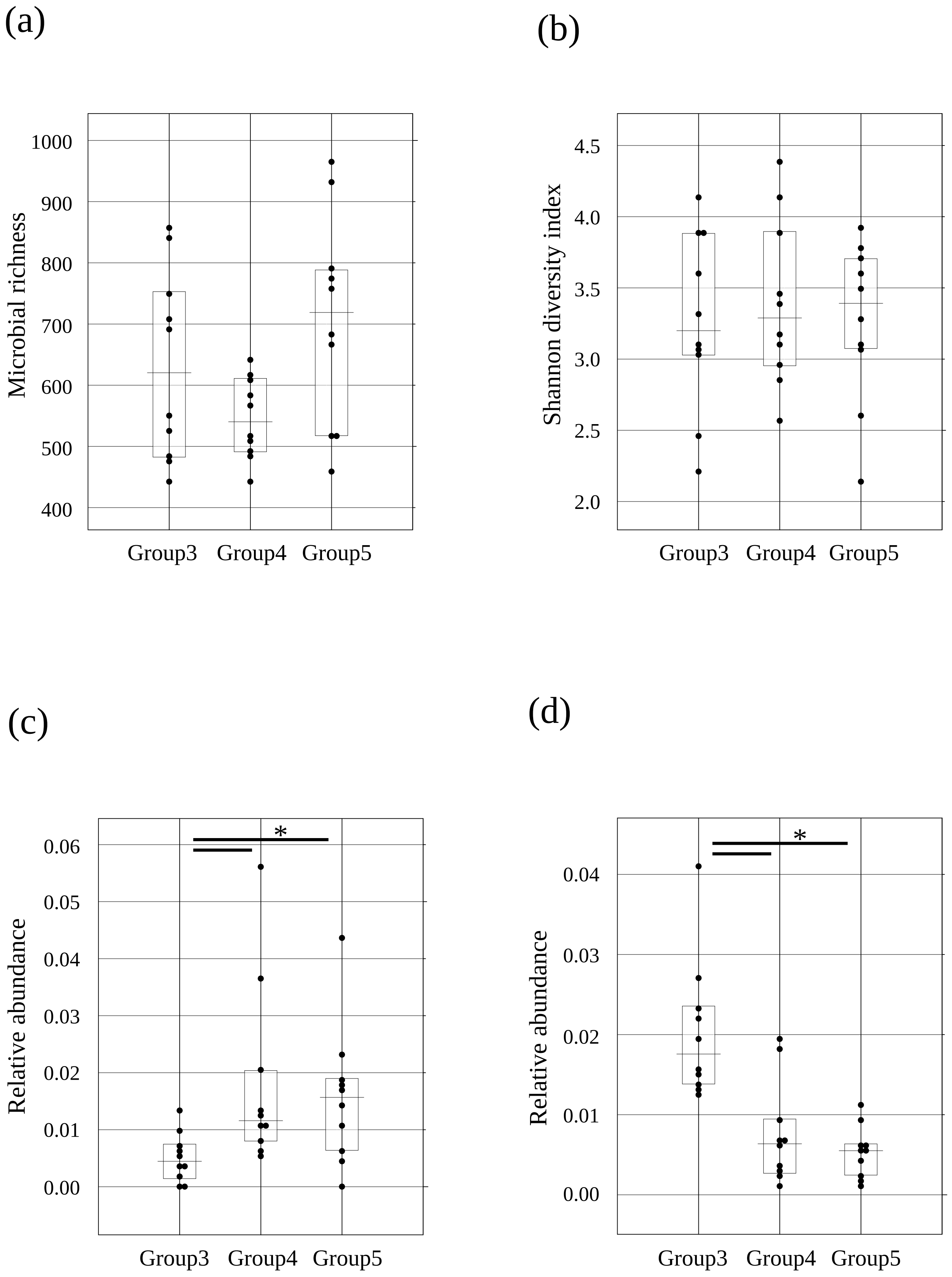

(a)
(b)
(c)
(d)
Microbial richness
1000
900
800
700
600
500
400
Group3
Group4
Group5
Shannon diversity index
4.5
4.0
3.5
3.0
2.5
2.0
Relative abundance
0.06
0.05
0.04
0.03
0.02
0.01
0.00
*
0.04
0.03

Figure S5

Table S1. Changes in the fecal microbiota between lantibiotic positive and negative groups based on taxon-based analysis

	Unassigned;Other	Actinobacteria	Bacteroidetes	Cyanobacteria	Firmicutes	Fusobacteria	Lentisphaerae	Planctomycetes	Proteobacteria	Synergistetes	TM7	Tenericutes	Verrucomicrobia
G1	1.31E-03	5.71E-02	3.68E-01	3.93E-06	4.26E-01	3.00E-03	4.33E-05	0.00E+00	1.35E-01	0.00E+00	4.94E-05	1.88E-05	8.70E-03
G2	9.79E-04	7.49E-02	3.41E-01	4.66E-06	5.15E-01	9.56E-04	0.00E+00	3.84E-07	5.30E-02	8.50E-06	2.27E-05	4.30E-06	1.46E-02

Group 1 (G1) : Lantibiotic positive group, Group 2 (G2) ; Lantibiotic negative group

Table S2. Changes in the fecal microbiota between Mutacin I/III or Smb positive and negative groups based on taxon-based analysis

	Unassigned;Other	Actinobacteria	Bacteroidetes	Cyanobacteria	Firmicutes	Fusobacteria	Lentisphaerae	Planctomycetes	Proteobacteria	Synergistetes	TM7	Tenericutes	Verrucomicrobia
G1a	1.49E-03	7.50E-02	3.32E-01	3.29E-06	3.89E-01	5.09E-03	0.00E+00	0.00E+00	1.89E-01	0.00E+00	4.48E-05	0.00E+00	8.96E-03
G1b	1.19E-03	4.58E-02	3.91E-01	4.33E-06	4.49E-01	1.69E-03	7.03E-05	0.00E+00	1.02E-01	0.00E+00	5.22E-05	3.05E-05	8.55E-03
G2	9.79E-04	7.49E-02	3.41E-01	4.66E-06	5.15E-01	9.56E-04	0.00E+00	3.84E-07	5.30E-02	8.50E-06	2.27E-05	4.30E-06	1.46E-02

Group 1a (G1a) : Mutacin I positive group, Group 1b (G1b) ; Smb I positive group, Group 2 (G2) ; Lantibiotic negative group

Table S3. Salivary microbiota taxon-based analysis

	Unassigned;Other	Other	Actinobacteria	Bacteroidetes	Chlamydiae	Cyanobacteria	Firmicutes	Fusobacteria	GN02	Planctomycetes	Proteobacteria	SR1	Spirochaetes	Synergistetes	TM7	Tenericutes	Verrucomicrobia
G1	7.27E-03	1.59E-06	1.37E-01	5.40E-02	0.00E+00	1.22E-05	4.31E-01	2.09E-02	2.59E-04	1.49E-06	3.36E-01	1.08E-03	3.64E-04	1.44E-05	1.14E-02	6.46E-06	5.87E-05
G2	0.008498	5.13E-06	0.127662	0.084737	2.71E-07	0.000121	0.503015	0.021748	0.000224	8.7E-07	0.245839	0.001193	0.000144	6.78E-06	0.00673	1.55E-05	6.03E-05

Group 1 (G1) : Lantibiotic positive group, Group 2 (G2) ; Lantibiotic negative group

Table S4. Mouse fecal microbiota taxon based analysis

	Unassigned;Other	Actinobacteria	Bacteroidetes	Firmicutes	Fusobacteria	Proteobacteria	Synergistetes	TM7	Tenericutes	Verrucomicrobia
G3	7.28E-04	1.64E-03	8.75E-01	1.09E-01	1.82E-04	1.30E-02	1.34E-05	2.86E-04	1.14E-04	8.84E-05
G4	2.82E-04	1.41E-03	7.86E-01	1.95E-01	4.20E-05	1.52E-02	0.00E+00	6.96E-05	1.19E-05	2.26E-03
G5	0.000248	0.001342	0.808459	0.174421	2.74E-05	0.014221	0	0.000259	0.000562	0.00046

Group 3 (G3) : mouse fecal microbiota fed with *S. mutans* supernatant containing Mutacin I group, Group 4 (G4) ; mouse fecal microbiota fed with *S. mutans* supernatant without Mutacin I group, Group 5 (G5) ; mouse fecal microbiota fed with medium only I group
